# 

**Published:** 1911-01-21

**Authors:** 


					11
THE HOSPITAL January 21, 1911.
By Sir WILLIAM BENNETT, K.C.V.O., F.R.C.S.
RECENTLY PUBLISHED. Crown 8vo. 5s. net.
INJURIES AND DISEASES OF
THE KNEE JOINT.
With 34 Illustrations.
KISBET & C O -
Also, 8vo. 6s. FOURTH EDITION.
MASSAGE AND MOVEMENTS IN
RECENT FRACTURES;
Sprains and their Consequences ; Rigidity of
the Spine and the Management of Stiff Joints.
With 23 Illustrations.
Xj O UST <3- IM .A. it s , GBEEU CO.
A MANUAL OF
MEDICAL EXERCISES.
By PERCY LEWIS, M.D., M.R.C.S.,
Hon. Medical Officer to Victoria Hospital, Folkestone.
66 pages, with 35 Illustrations, cloth, Is. 6d. net; post free, Is. 7d.
H. K. LEWIS, 136 GOWER STREET, LONDON, W.C.
Just Issued. Ninth Edition, Rewritten and Enlarged, 9s. net.
PHARMACY, MATERIA MEDICA & THERAPEUTICS.
Being a Commentary on the B.P. and its Drugs, with their Preparations,
and on all the new Non-official Remedies in use, with Chapters on the
different processes used in Dispensing, Prescription-writing, Latin
Glossary, &c. By Sir W. WHITLA, M.D., LL.D., Professor, Materia
Medica, Queen's University, Belfast, and Senior Physician, Royal Victoria
Hospital.
By the same Author. New Work. 1,900 pages. Crown 8vo. 25s. net.
PRACTICE OF MEDICINE.
Containing in two Handy Volumes a complete and independent Article on
the Etiology, Symptoms, and Diagnosis of every Medical Disease; arranged
in Dictionary Form for Practitioners and Students.
London: BAnxiiRB, Tindaix & Cox, 8 Henrietta Street, W.C.
FIFTH EDITION. With 50 Illustrations. 51- net.
THE SCHOTT METHODS OF THE TREATMENT
OF CHRONIC DISEASES OF THE HEART.
With an Account of the Nauheim Baths and of their Therapeutic Exercises.
By W. BEZLY THORNE, M.D., M.R.O.P.
London : J. & A. Churchill, 7 Great Marlborough Street.
By A. H. TUBBY, M.S.
DEFORMITIES.
Six hundred pages, 300 Illustrations. Price 17s.
"Standard work on the subject in the English language."
British Medical Journal.
SURGERY OF PARALYSES.
By A. H. TUBBY, M.S.Lond., F.R.C.S.Eng., and
ROBERT JONES, F.R.C.S.E.
Illustrated by 93 figures and 68 cases. Pp. 301. 10s. net.
" Can confidently recommend it as a practical summary of the most
modern views."?Edinburgh Medical Journal.
" Indicates original and successful methods of treatment."
American Journal of Orthopaedic Surgery.
London : Macmillan & Oo., Ltd. New York : The Macmillan Oo.
A NEW BOOK. Crown 8vo. 5s. net.
THE NEWER REMEDIES (a Practical Guide To).
By J. M. Fortescue-1 shickdale, M.A., M.D. Oxon., Phys., Olifton
Coll.; Asst. Pliys., B'tol Roy. Infy.; Lect. Pharm., Univ. of Oxford ; Clin.
Lect., Univ. of Bristol. An account of the Principal New Drugs, with
indications as to their value and employment.
" Will help the harassed practitioner."?Hospital.
" We very cordially recommend it."?South African Medical Record.
Bristol: J. Wright & Sons, Ltd. London : Simpkin & Co., Ltd.
THERAPEUTICS OF THE CIRCULATION
By Sir LAUDER BRUNTON, Bart., M.D., D.Sc., F.R.C.P., fee.,
Consulting Physician to St. Bartholomew's Hospital.
Published under the Auspices of the University of London.
With numerous Diagrams. Medium 8vo. 7s. 6d. net.
JOHN MURRAY, Albemarle Street, W.
The memory of Sister Madge Kelly, principal of
" Coonara " Private Hospital, Melbourne, who died a few
?weeks ago, is to be perpetuated by a suitable memorial.
So resolved a meeting held on November 22, largely
attended by the medical profession, nurses, and friends.
The form of the memorial is left open for the present.
This trained nurse, who followed private practice for
many years, was of so cheerful and courageous a disposi-
tion, and so strong a personality, that it was commonly
said she was better than doctor or physic to her patients,
and she has frequently, as it seemed, plucked the 6ick
back from the very brink of the grave.
MEDICAL APPOINTMENTS, ETC.
At the monthly meeting of the Board of Management
of the Bradford Royal Infirmary, held on Friday, Janu-
ary 6, Mr. James Phillips, F.R.C.S. Edin., was appointed
honorary surgeon to the institution to fill the vacancy on the
honorary staff caused by the death of Mr. W. Horrocks,
F.R.C.S. Mr. Phillips has been senior honorary assistant;
surgeon for the last five years.
The following officers have been reappointed at the'
Manchester Royal Infirmary to be anaesthetists : Mr. S. R.
Wilson, M.B., B.S. Lond., F.R.C.S. Edin.; Mr. R. A. M.
Atkinson, M.B., Ch.B. Vict, j and Mr. T. Coogan, M.B.,
Ch.B. Vict. To be medical officer for radiography and
electricity department : Mr. A. E. Barclay, M.B., B.C.
Cantab., M.R.C.S., L.R.C.P. To be director of the clinical,
laboratory : Mr. G. E. Lovedav, M.B., B.C. Cantab.,.
M.R.C.S., L.R.C.P.
In consequence of the resignation of Professor M. M.
McHardy, who for many years was senior ophthalmic
surgeon to King's College Hospital, Mr. L. Vernon Cargill,
F.R.C.S., has been promoted to the post of senior ophthal-
mic surgeon, and Mr. H. Willoughby Lyle, M.D.,
B.S.Lond., F.R.C.S., has been appointed assistant ophthal-
mic surgeon.
The King has approved of the appointment to the con-
sulting staff of the King Edward VII. Sanatorium, Mid-
hurst, of Dr. Theodore Dyke-Acland, M.D., F.R.C.P., and
Dr. St. Clair Thomson, M.D., F.R.C.P., F.R.C.S.
Dr. A. L. Baly, assistant medical officer of the Lambeth
Infirmary, was yesterday "appointed by the Board of
Guardians chief medical officer.
The Duke of Connatjght has appointed Lieut.-Colonel
Sir James Richardson Andrew Clark, C.B., F.R.C.S.E.,
Chief Commissioner of the St. John Ambulance Brigade,
in place of Inspector-General Belgrave Ninnis, R.N.,
resigned.
The Victorian Odontological Society held its annual
Congress for one day only this year on November 22. The
Dental College was open the whole day for free treatment,,
and some sixty to seventy people availed themselves of the
privilege.
THE MEDICAL SCHOOLS.
The following are the arrangements for next week at the
Medical Graduates' College and Polyclinic, 22 Chenies
Street, Gower Street, W.C. : Monday, January 23. at
4 p.m., Mr. J. E. It. McDonagh, Clinique (Skin) ; at
5.15 p.m., Dr. F. J. McCann, " The Treatment of Puer-
peral Infection." Tuesday, at 4 p.m., Dr. C. 0. Haw-
thorne, Clinique (Med.); at 5.15 p.m., Dr. Harry Camp-
bell, "The Neurone Theory." Wednesday, at 4 p.m.,
Mr. Edred M. Corner, Clinique (Surg.); at 5.15 p.m., Dr.
Nestor Tirard, " Dyspnoea in Bright's Disease and in
Diabetes." Thursday, at 4 p.m., Dr. Leonard Guthrie,
Clinique (Med.); at 5.15 p.m., Dr. T. D. Lister, "Pro-
gnosis in Phthisis " (second lecture). Friday, at 4 p.m.,
Mr. E. B. Waggett, Clinique (Ear, Nose, and Throat).
The following are the arrangements for next week at the
West London Postgraduate College : At 10 a.m ,
January 23 and 26, demonstration by Surgical Registrar;
at 10 a.m., January 25, gynaecological demonstration; at
11.30 a.m., January 24, demonstration of minor operations ;
at 12 noon, January 23, pathological demonstration; at
12.15 p.m., January 26, lecture, Dr. Grainger Stewart,
" Practical Medicine." Lectures at 5 p.m. : January 23,
Mr. Armour, " Operations for Gall Stones" ; January 24,
Dr. Saunders, clinical lecture with cases ; January 25, Dr.
Beddard, "Practical Medicine" (lecture 2); January 26,
Mr. Baldwin, "Practical Surgery" (lecture 2);
January 27, Mr. Rickard Lloyd, " Ana?sthetics."
BOOKS RECEIVED.
Lippincott.
" International Clinics." Vol. III., twentieth series. 1910.
Cable Printing and Publishing Co.
"Amateur Gardener's Diary and Dictionary," 1911.
Cassell and Co.
" Diseases of the Joints and Spine." By H. Marsh and
C. G. Watson.
A. and C. Black.
" Black's Medical Dictionary." New edition. By J. D.
Comrie.
" Who's Who, 1911."
" The Writer's and Artist's Year-book, 1911."
" The Englishwoman's Year-book, 1911." By Mitson.
Methuen.
" Preliminary Physiology." By W. Narramore.
Oliver and Botd.
" Food and Feeding in Health and Disease." By C. Watson.
J. Wright and Son.
" Dispensing Mado Easy." By W. G. Sutherland, revised
by F. J. Warwick. Fourth edition.
" An Introduction to Surgery." By R. Morrison.
" Golden Rules for Diseases of Children." By E. B. Smith.
Gifts and Bequests.
The Queen has sent her annual subscription of five
guineas to the British Home and Hospital for Incurables,
Streatham. Queen Alexandra has also sent her annual
.subscription of five guineas.
Among the latest contributions to King Edward's Hos-
pital Fund for London is the sum of ?1,000, being the
annual subscription of his Majesty the King, Patron and
late President of the Fund, and the sum of ?105, being
the annual subscription of her Majesty the Queen.
The Leicester Infirmary has received a donation of ?105
from the family of the late Mr. Samuel Harding, in
memory of their father, and the Board have'recommended
tho appointment of Messrs. W. W. Harding and E. W.
Harding, his two sons, as Life Governors of the Infirmary.
The institution has also received a sum of ?21 as the share
of the recent performances by the New Leicester Amateur
Operatic Society.
Prince Alexander of Teck has received the following
contributions to the Prince Francis of Teck Memorial Fund
for the endowment of Middlesex Hospital : Miss Helena
Finnie, ?40; Lord Allendale, ?25; Miss Emily C. Druce,
?10; Mr. and Mrs. H. V. Higgins, ?10; Sir John
Pound, Bart., ?5; ?500 from Mr. R. Nivison in addition
to the ?500 which Mr. Nivison sent last June to the late
Prince Francis to relieve the hospital from its debt;
Mrs: M. J. Gooch and Mr. Graham Prentice, ?25 each;
Mr. W. Asch, ?21; La Marquisa Yinda de Misa, Major
H. Donald M. Leile, and the Vintners' Company, ?10 10s.
each; and Colonel Charles Needham, ?5 5s. ; and
an annual subscription of ?100 from Mrs. William
Milburn.
The executors of the will of the late Louisa Sophia Lady
GoTdsmid now make a further distribution of ?9,700, re-
presenting part of her residuary estate, and have selected
charities, numbering .about thirty in all, for participation
in this sum. To the following general charities have been
allotted sums of ?500 : Hospital for Women, Soho Square ;
St. Mary's Hospital for Women and Children, Plaistow;
East London Hospital for Children and Dispensary for
Women; Queen's Hospital for Children; the Hospital for
Invalid Gentlewomen; Mount Vernon Hospital for Con-
sumption and Diseases of the Chest; Royal Waterloo
Hospital for Children and Women; Paddington Green
Children's Hospital; and Royal Free Hospital. The
Jewish charities benefited include the Jewish Board of
Guardians (for the endowment of two more beds at their
new Convalescent Home), ?1,300.
Lord Sandhurst, Treasurer of St. Bartholomew's Hos-
pital, acknowledges the receipt of ?200 from " H. P."
Lady Juliet Duff has received from Mr. R. Nivison
?500 towards the fund of ?100^)00 which.jjhe is raising
on behalf of Charing Cross Hospital.
Mr. John Bonnett, of Regent Street, Cambridge,
solicitor, who left estate valued ai ?16,370 gross, has
bequeathed ?200 to Addenbrooke's Hospital, Cambridge.
Mr. William Strang Steel, of Philiphaugh, Selkirk-
shire, has bequeathed r,o the Western Infirmary of Glas-
gow ?5,000 and to the Royal Infirmary, Edinburgh,
?2.500.
Mrs. Alexandrina Dunn Saunders, of Winchfield,
Hants, has bequeathed ?100 to the Hospital for Nervous
Diseases, Welbeclc Street, and ?50 to the Surgical Aid
Society.
Mr. Nivison has sent donations of ?500 each to the
general funds of University College Hospital, the West-
minster Hospital, and the Brompton Hospital for Con-
sumption, and a further donation of ?500 to St. Mary's
Hospital, Paddington.
The committee of management of King's College Hos-
pital have received ?500 from Mr. R. Nivison. and ?100
from .Lord Ashcombe, in connection with the Ladies'
Dinner to be held next May on behalf of the fund for the
removal of the hospital to South London.
Mr. Joseph Boden, of Cheshire, silk merchant, has be-
queathed ?200 each to the Manchester Royal Infirmary
and Dispensary, the Manchester Southern Hospital for the
Diseases of Women and Children, the Manchester Clinical
Hospital for the Diseases of Women and Children, and to
St. Mary's Hospital and Dispensary for the Diseases of
Women and Children.
Mrs. Rachel Mocatta, of Albert Hall Mansions, Ken-
sington, has, subject to specific bequests, left the residue
of her estate upon trust for her son and his children,
directing that any surplus income not used for their main-
tenance for a period of twenty-one years after her death
shall be paid to University College Hospital. On the
death of her son she bequeathed ?2,000 to University Col-
lege Hospital for the endowment of a bed in memory of
her brother, George Goldsmid ; and on the death of the
survivor of her son and his children the ultimate residue
of her estate as to one-fourth to the Hospital for Sick
Children, Great Ormond Street, and one-fourth to the
Cancer Hospital, Fulham.
There is now issued the report, dated November 30,
1910, of the sub-committee appointed by the Executive
Committee of King Edward's Hospital Fund for London
to consider the question of co-operation in respect of hos-
pital contracts, of which Mr. John G. Griffiths was chair-
man. The advantages of co-operative purchase by groups
of independent voluntary hospitals, and the possibilities
of economy in that direction, were discussed by the Duke
of Teck at the meeting of the Governors and Genex-al
Council of the Fund on December 14 last. His speech
was reported in full in The Hospital of December 17
. (page 362). . .
Queen Alexandra has forwarded to the committees of
the British Home and Hospital for Incurables and of the
Gordon Boys' Home cheques amounting to ?11 18s. 2d.,
the sum resulting from the sale during the past year of the
late Canon Fleming's sermon "Recognition in Eternity."
This sermon, published by Messrs. Skeffington and Son,
of 34 Southampton Street, Strand, was preached at Sand-
ringham Church on January 24, 1892, and has realised a
profit of ?1,724 14s. 2d., which her Majesty has divided in
equal parts between the two institutions mentioned above.
Jottings.
In connection with the Eastbourne District of the
League of Mercy a theatrical performance will be held at
the Devonshire Park Theatre at 2.30 on Tuesday, Janu-
ary 24.
The Liverpool Florence Nightingale Fund has reached
about ?3,000, and will be transferred to the Liverpool
Queen Victoria District Nursing Association to establish
and maintain a Florence Nightingale Home for district
nurses.
Miss Jane Hardy, who has been a pensioner of the
Royal Hospital for Incurables, Putney Heath, for the long
period of forty years, is being admitted as an in-patient
owing to the invalid's increasing weakness and other
adverse circumstances.
The King Edward Memorial Committee for Lynn and
West Norfolk has decided to use its funds in improving
the entrance to the West Norfolk and Lynn Hospital,
erecting a new staircase, and placing a bust of his Majesty
above it. The amount subscribed up to the present is
about ?1,300.
The accounts of the Hospital Saturday Fund for 1910
closed last week. The total receipts reach ?34,731, being
over ?4,000 in excess of the income for 1909. The secre-
tary, Mr. A. W. Davis, stated that if to this income were
added the sums received by the Standing Committee, the
total income would amount to ?42,359.
The sum of ?9,681 has been subscribed for the Norfolk
memorials to King Edward. Of this sum ?329 has been
devoted to special purposes, including a bronze bust of the
late King at the Norfolk and Norwich Hospital; and the
balance, less expenses, is to be applied to the endowment
of the Norfolk and Norwich Hospital extension buildings.
In the eighteenth century the administrators of the
Hopital de la Charite of Lyons found themselves com-
pelled, in order to prevent patients going in and out of
the hospital, to tolerate in the establishment a sort of
tavern or cellar, to which only the inmates of the house
co'uid come to buy. A sister was placed in charge of it,
and had two girls of the house under her command to deal
with customers; that is to say, two orphans brought
up at the Charite. The sister received six livres every
six months; the servants, whose work was heavier,
nine livres each : that is to say, thirty sous per month.
The wine was supplied from the cellars of the establish-
ment, and the sister had each month to pay over her
receipts into th? hospital chest, and to give an account
of the wine sold and of that which still remained in stock.
For each anee (93 litres .222, as much as an ass was sup-
posed to be able to carry) she had to give to the Eector
17 livres 12 60U6. Wine was 6old at 4 sous a pot. In
1761 the annual consumption was 47,904 pots, or, roughly,
130 a day, equivalent to 137 litres daily, or 501 hectolitres
per year. Many an ordinary tavern had a far smaller
sale. The profits from the cellar must have worked out :.t
about 5,000 livres a year.
TO HOSPITAL SECRETARIES AND OTHERS.
We invite contributions from any of our readers, and
shall be glad to pay, according to length, for items of news
for the institutional section (including appointments, resig-
nations, gifts, etc.) which we may publish. All payments
for manuscripts used are made as early as possible after
the beginning of each quarter.
Obituary.
Dr. Thomas James has died in London at the age of
fifty-four. Dr. James took his L.S.A. in 1878, his-
M.R.C.S. in 1879, and his L.R.C.P. in 1881. He studied
at the Middlesex Hospital and became an F.R.C.S. irt
1885; then completed his education at Durham University,,
where he graduated M.D. in 1890. His appointments
included those of surgeon to the Central London Oph-
thalmic and clinical assistant at the Royal London
Ophthalmic Hospitals. He had been also house surgeon,,
house physician, and resident obstetrician at the Middle-
sex.
The death, resulting from a fall, of Dr. John Coombe-
Maddever, aged sixty-one, of Coombe House, Brownhills,
Walsall, Staffordshire, is announced. Dr. Maddever was
educated at Glasgow University, where he took his M.B.
and C.M. in 1872, and graduated M.D. two years later.
His appointments included that of medical officer for the
Brownhills district, and senior surgeon at Hammerwich
Hospital and to the Old and New Brownhills and other
collieries.
THE WEEK'S NOVELTIES.
During the winter months many people fmd it advan-
tageous to be possessed of good, reliable HOT-WATER
BOTTLES. It is not always easy to secure these ser-
viceable and comforting articles; but nowadays, when :j<>
one really bothers about a warming-pan (and not merely
disregards that old-fashioned apparatus in the Pick-
wickian sense), it is imperative that every housewife
should see to it that she secures the best article on the.
market. Andersons, Ltd., of 37 Queen Victoria Street,,
supply excellent rubber water bottles, which are cheap
and thoroughly reliable/ The firm has made such bottles,
for many years now, and many hospitals and institution;*
have found the rubber "Andersons" the best of the r
kind. An institutional hot-water bottle must stand rough
treatment to a degree which the domestic hot-water bottle
cannot possibly endure; it is a high testimonial to the
merits of Andersons' goods that they have so generally
eat'sfied hospital authorities.
As a complete first-aid dressing VULNOPLAST, which
is manufactured by the Liverpool Lint Co. (Mark Street-
Mills, Liverpool), is well worth a trial. It is simple and
easy to apply, and demands neither pins nor bandages. It
is eminently a household necessity; and every medicine
cupboard should contain a supply.
In bronchial and laryngeal troubles inhalation is one of
the most soothing and satisfying methods of giving relief
to the patient's distress. There are many compounds for
vaporising purposes sold by chemists, and of that number
VAPO CRESOLENE, made by the well-known firm of
Allen and Hanburys, is specially useful. It is an agreeable
preparation, the fumes given off being pleasant and un-
irritating, while their germicidal power is very great. In
the treatment of children's pulmonary conditions it is par-
ticularly to be recommended.
The ventilators manufactured by Robert Boyle and Son,,
of Holborn Viaduct, are well known to sanitary engineers.
The latest model, BOYLE'S AIR PUMP VENTILATOR,
is well adapted for institutional use. It possesses no com-
plicated parts likely to get out of order, and requires no-
attention once it has been fixed. As a ventilator it is
highly efficient. The patent has secured many gold anqii
silver medals and diplomas of merit.
THE FUTURE T??'E HOSPITALS
MEDICAL RELIEF FOR ALL ACCORDING TO MEANS.
A Scheme of Co-operation between the PATIENTS, the HOSPITALS,
and the STATE.
Price 2d. By Sir HENRY BURDETT, K.C.B., K.C.V.O. Price 2d.
THE SCIENTIFIC PRESS, Ltd., 28 & 29 Southampton ? St., Strand, London, W.C.,
and at all Bookstalls.

				

